# Denitrifying Bacterial Communities Affect Current Production and Nitrous Oxide Accumulation in a Microbial Fuel Cell

**DOI:** 10.1371/journal.pone.0063460

**Published:** 2013-05-23

**Authors:** Ariadna Vilar-Sanz, Sebastià Puig, Arantzazu García-Lledó, Rosalia Trias, M. Dolors Balaguer, Jesús Colprim, Lluís Bañeras

**Affiliations:** 1 Molecular Microbial Ecology Group, Institute of Aquatic Ecology, Universitat de Girona, Girona, Spain; 2 LEQUIA, Institute of the Environment, Universitat de Girona, Girona, Spain; Graz University of Technology (TU Graz), Austria

## Abstract

The biocathodic reduction of nitrate in Microbial Fuel Cells (MFCs) is an alternative to remove nitrogen in low carbon to nitrogen wastewater and relies entirely on microbial activity. In this paper the community composition of denitrifiers in the cathode of a MFC is analysed in relation to added electron acceptors (nitrate and nitrite) and organic matter in the cathode. Nitrate reducers and nitrite reducers were highly affected by the operational conditions and displayed high diversity. The number of retrieved species-level Operational Taxonomic Units (OTUs) for *narG*, *napA*, *nirS* and *nirK* genes was 11, 10, 31 and 22, respectively. In contrast, nitrous oxide reducers remained virtually unchanged at all conditions. About 90% of the retrieved *nosZ* sequences grouped in a single OTU with a high similarity with *Oligotropha carboxidovorans nosZ* gene. *nirS*-containing denitrifiers were dominant at all conditions and accounted for a significant amount of the total bacterial density. Current production decreased from 15.0 A·m^−3^ NCC (Net Cathodic Compartment), when nitrate was used as an electron acceptor, to 14.1 A·m^−3^ NCC in the case of nitrite. Contrarily, nitrous oxide (N_2_O) accumulation in the MFC was higher when nitrite was used as the main electron acceptor and accounted for 70% of gaseous nitrogen. Relative abundance of nitrite to nitrous oxide reducers, calculated as (q*nirS*+q*nirK*)/q*nosZ*, correlated positively with N_2_O emissions. Collectively, data indicate that bacteria catalysing the initial denitrification steps in a MFC are highly influenced by main electron acceptors and have a major influence on current production and N_2_O accumulation.

## Introduction

Nitrogen contamination is one of the main concerns in wastewater treatment. The complete elimination of nitrogen from water involves many metabolic steps, which are catalysed by microorganisms with complex metabolic requirements [1]. Biological treatments have provided practical solutions to these difficulties and have become reliable and highly cost-effective methods for nitrogen removal in wastewater treatment plants (WWTPs). Ammonium is the major nitrogen compound in wastewater, and sequential aerobic and anaerobic processes are needed to ensure its complete removal using a coupled nitrification-denitrification process. However, in wastewater exhibiting a low carbon to nitrogen ratio, the conventional heterotrophic process of denitrification becomes challenging. This carbon dependence reinforces the need for the development of autotrophic processes to avoid the additional costs involved in adding organic matter to water treatments [2]. Denitrifying microbial fuel cells (MFCs) are among the most promising technologies that are currently used to partially suppress the carbon dependence of denitrification [3], [4], [5]. In a MFC, organic substrates are oxidised by exoelectrogenic bacteria, which produce electrons that are transferred to an anode electrode and then flow to a cathode. The anode and cathode are linked by a conductive material containing a resistor. Protons produced at the anode migrate through the solution across a cation exchange membrane to the cathode chamber where electrotrophic bacteria combine protons with a reducible compound and electrons.

Complete denitrification, using either nitrate or nitrite as electron acceptors, has been achieved using biocathodic bioelectrochemical systems [3], [4], [5], [6], [7]. Chemolithoautotrophic denitrifiers regulate the denitrification process in the cathode of MFCs when no organic matter is present, similarly to what have been proposed for nitrogen-contaminated groundwater [8], [9]. Nitrate reduction on the cathode in a microbial fuel cell requires significantly lower influent COD/N ratios [5] than for traditional nitrogen removal in wastewater treatment plants. In the latter, COD/N ratios between 7 and 10 are typically required [10]. Virdis *et al*. [5] found that the ratio between the COD removed at the anode and the nitrate removed at the cathode, was equal to 4.48 g COD g^−1^ NO^3–^N.

Previous studies have shown that the cathodes of MFCs harbour complex bacterial communities, including members of *Proteobacteria, Firmicutes* and *Chloroflexi* phyla as the most abundant species [11], [12], [13]. Wrighton *et al.* [13] showed that the observed changes in the dominant members of the bacterial community did not correspond with the changes in reactor functioning. This observation potentially reflects the fact that most of the observed phylotypes did not exhibit relevant denitrification activity, suggesting that information about functional groups is required for a better understanding of the process.

Denitrification is an anaerobic respiration pathway catalysed by taxonomically diverse bacteria and archaea [14]. Thus, molecular studies are often directed to the use of functional genes [15]. The commonly used markers for nitrate reduction include either membrane-bound (Nar type) or soluble periplasmic (Nap) nitrate reductases, encoded by *narG* and *napA* genes, respectively [14], [16], [17]. Dissimilatory nitrite reductases (Nir) exist as two functionally equivalent enzymes: a cytochrome *cd_1_*-type and a copper containing encoded by the *nirS* and *nirK* genes, respectively, which have been thoroughly used as molecular markers for denitrification [18], [19], [20], [21]. The reduction of nitrous oxide that occurs during the last step in the denitrification pathway has received most of the attention in molecular studies. Nitrous oxide reductase mediates the production of nitrogen gas and is coded by the *nosZ* gene [14], [22].

The sequential nature of the denitrification process, in which the products of a reaction serve as substrates for the next reduction step, makes this process an excellent example to study cooperation through electron transfer between different bacterial cells. The direct electron transfer between cells of different species has been demonstrated in the anodes and cathodes of MFCs [23], [24]. In fact, the functioning of MFCs partly relies on the capacity of microorganisms to accept electrons from the cathode directly via membrane bound cytochromes or indirectly via added (exogenous) or secreted (endogenous) mediators [25].

Our goal was to identify the relevant players of nitrate, nitrite and nitrous oxide reductions as key metabolic steps in the denitrification process in the biofilms generated in the cathode of a MFC at different operating conditions. The different conditions were determined in order to assess the effect of different electron donors (electron transfer between the electrode or organic matter) and different electron acceptors (nitrate or nitrite) in the microbial community. We examined the community composition and abundance of nitrate, nitrite and nitrous oxide reducing bacteria using five functional genes of the denitrification pathway. Abundances of functional genes were analysed through quantitative PCR, and the structure of the nitrate reducer, nitrite reducer and nitrous oxide reducer bacterial communities were assessed using a cloning-sequencing approach. MFC performances, in terms of nitrogen removal, power density generation and nitrous oxide accumulation, were compared for the different electron acceptors and donors used and related to prevailing denitrifiers.

## Materials and Methods

### Experimental Set-up

The MFC consisted of an anode and a cathode placed on opposite sides of a single methacrylate rectangular chamber (dimensions, 29×26×400 mm; empty bed volume of 265 mL and 385 mL for anode and cathode chambers, respectively). The anode and cathode chambers were filled with rod graphite (Alfa Aesar, Germany), which reduced the compartment volumes to 120 and 145 mL (net anodic and cathodic compartments, NAC and NCC), respectively. Two thinner graphite electrodes (Sofacel, Spain) were connected to an external resistor (100 Ω) to close the electric circuit. A cation exchange membrane (CEM, Nafion 117, Dupont) was placed between the anode and cathode frames. The medium was continuously fed into a recirculation loop to maintain well-mixed conditions and avoid concentration gradients. The system was thermostatically controlled at 23±2 °C. Prior to treatment, the MFC was inoculated with 50 mL of effluent from the anode of a parent MFC [26] that was previously used to treat synthetic wastewater primarily composed of sodium acetate and a buffer solution.

### Microbial Fuel Cell Operation

The denitrifying MFC was operated for one year to treat acetate-enriched wastewater in the anode. The biocathode was fed with different electron acceptors (nitrate and nitrite) in two different media (mineral media and effluent from an air-cathode MFC treating urban wastewater). Table 1 shows the main influent characteristics of the anode and cathode compartments during the experimental period.

**Table 1:** Chemical characteristics of the anode and cathode influents of the denitrifying MFC.

		Anode		Cathode			
Period	Days ofoperation	Flow (L·d^−1^)	COD (mg·L^−1^)	Flow (L·d^−1^)	COD (mg·L^−1^)	N-NO_2_^−^ (mg·L^−1^)	N-NO_3_^−^ (mg·L^−1^)
**Period 1** (Autotrophic with NO_3_^−^)	0–76	2.4±0.3	745±231	2.0±0.3	n.d.	0.3±0.2	26.6±1.3
**Period 2** (Heterotrophic with NO_3_^−^)	77–182	1.5±0.6	895±175	1.4±0.5	64±21	1.0±0.4	29.6±5.3
**Period 3** (Autotrophic with NO_2_^−^)	183–350	1.2±0.2	923±266	1.2±0.2	n.d.	20.3±1.7	2.9±4.7

Represented values are the means and standard deviations (n = 5) at steady state conditions.

COD: chemical oxygen demand, N-NO_2_^−^: nitrogen in form of nitrite; N-NO_3_^−^: nitrogen in form of nitrate; n.d., not detected.

To determine the influence of the electron acceptor on the MFC performance and the cathode community dynamics, the experimental study was divided into three different periods. Period 1 was operated at constant conditions for 76 days, period 2 for 105 days, and period 3 for 167 days. The cathode was fed differently depending on the operational period. In periods 1 and 3, the enriched mineral medium contained 0.488 g L^−1^ NaHCO_3_, 0.2 g L^−1^ NaNO_3_, 0.92 g L^−1^ NaH_2_PO_4_ 2H_2_O, 0.0056 g L^−1^ CaCl_2_ 2H_2_O, 0.036 g L^−1^ MgSO_4_·7H_2_O, 0.0052 g L^−1^ KCl and 0.1 mL L^−1^ of a microelements solution (SL10). The nitrate substrate was added in period 1 as an electron acceptor that was subsequently replaced by nitrite in period 3. These periods are hereafter respectively referred to as autotrophic with nitrate and autotrophic with nitrite. During period 2, the effluent from an air-cathode MFC typically used for treating urban wastewater was used [26]. This feeding condition is referred as heterotrophic with nitrate in the results section. During period 1, the cathode of the MFC was fed with 2.0±0.3 L·d^−1^ of nitrate-enriched mineral medium (26.6±1.3 mg N-NO_3_^−^·L^−1^). After 77 days of operation, 1.4±0.5 L·d^−1^ of the effluent from an air-cathode MFC used to treat urban wastewater was fed to the cathode of the MFC [26]. The effluent of the air-cathode MFC also contained residual organic matter (64±21 mg COD·L^−1^). This concentration was similar to that of the non-biodegradable organic matter in urban wastewater. The third period began on day 183 in which 1.2±0.2 L·d^−1^ of nitrite-enriched mineral medium (20.3±1.7 mg N-NO_2_^−^·L^−1^) was fed without any organic matter content.

The anodic feed consisted of a nitrogen-purged medium enriched with acetate. The medium contained 1.44 g L^−1^ NaCH_3_COOH; 0.488 g L^−1^ NaHCO_3_; 0.03 g L^−1^ NH_4_Cl; 0.92 g L^−1^ NaH_2_PO_4_·2H_2_O; 0.0056 g L^−1^ CaCl_2_·2H_2_O; 0.035 g L^−1^ MgSO_4_·7H_2_O; 0.0052 g L^−1^ KCl; 0.044 g L^−1^ NaNO_3_; and 0.1 mL L^−1^ of a microelements solution (1 g L^−1^ FeSO_4_·7H_2_O; 70 mg L^−1^ ZnCl_2_; 100 mg L^−1^ MnCl_2_·4H_2_O; 6 mg L^−1^ H_3_BO_3_; 130 mg L^−1^ CaCl_2_·6H_2_O; 2 mg L^−1^ CuCl_2_·2H_2_O; 24 mg L^−1^ NiCl_2_·6H_2_O; 36 mg L^−1^ Na_2_Mo_4_·2H_2_O; and 238 mg L^−1^ CoCl_2_·6H_2_O).

### Analyses and Calculations

Liquid-phase samples for organic matter (chemical oxygen demand, COD) and nitrogen (ammonium: N-NH_4_^+^; nitrite: N-NO_2_^−^ and nitrate: N-NO_3_^−^) were sampled regularly and analysed according to the Standard Methods for the Examination of Water and Wastewater [27]. The levels of nitrous oxide (N-N_2_O) production were estimated according to the electron balance at the cathode following the methodology of Virdis *et al.* [5]. Experiments carried out using liquid- and gas-phase N_2_O analysers demonstrated excellent fits between measured data and estimated data using the electron balance [28], [29]. The nitric oxide (NO) production was considered to be negligible. To close the mass balance, the level of dinitrogen gas in the effluent was calculated from the current produced according to the following equation.
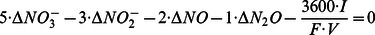



*ΔNO_3_^–^* is the nitrate consumption rate, whereas *ΔNO_2_^–^*, *ΔNO*, and *ΔN_2_O* are nitrite, nitric oxide, and nitrous oxide production rates, respectively. *I* is intensity, *V* voltage applied, and *F* the Faraday’s constant.

The cell potential (V) in the MFC circuit was monitored at one-minute intervals using an on-line multimetre (Alpha-P, Ditel) equipped with a data acquisition system (Memograph M RSG40, Endress+Hauser). The current (I) was calculated according to Ohm’s law. The current density was calculated by dividing the current by the net cathodic volume (A·m^−3^ NCC). The Coulombic efficiencies for nitrate and nitrite reduction were calculated according to Virdis *et al*. [5].

### Community Sampling and DNA Extraction

Two graphite rods (6×38 mm) were collected aseptically from different positions in the cathode chamber at steady state conditions at days 51 (autotrophic growth with nitrate), 106 (heterotrophic growth with nitrate) and 350 (autotrophic growth with nitrite) of operation. The rods were replaced with an equal number to maintain the working volume of the cathode chamber. The graphite rods were chilled on ice after collection and processed as independent samples for DNA extraction within two hours after sampling.

Bacterial biofilms were detached from the graphite rods using the following procedure. The rods were washed three times in Ringer solution (Sharlau®, Barcelona, Spain). Subsequently, the rods were immersed in 4 mL of 0.1 M of sodium pyrophosphate (Na_4_P_2_O_7_·10H_2_O), and the biofilm dislodged using three consecutive sonication rounds for 20 seconds followed by 30 seconds on ice. The suspended bacterial cells were pooled and centrifuged at 10,000 rcf for 2 minutes. The DNA was extracted using the FastDNA® SPIN Kit for soil (MP, Biomedicals) following the manufacturer’s instructions. The obtained DNA was quantified using a NanoDrop ND-1000 spectrophotometer (NanoDrop Technologies, Inc., Wilmington, DE) and stored at −20°C.

### Amplification and Quantification of Genes Using PCR

Five functional genes of the denitrification pathway (*narG*, *napA*, *nirS*, *nirK* and *nosZ*) were PCR amplified using primers and conditions previously published with minor modifications (Table S1). In all cases, the PCR reactions were performed in a total volume of 50 µL containing: 1X PCR buffer, 0.2 mM of each deoxynucleotide triphosphate and 1 U of Taq polymerase. All chemicals and reagents were obtained from Qiagen (Qiagen, Germany). The PCR amplifications were performed in a Gene Amp® 2700 thermal cycler (Applied Biosystems). The correct size of amplification products was verified using electrophoresis on a 1.5% agarose gel and visualised through ethidium bromide staining.

The gene abundances of denitrifying bacteria were determined using quantitative PCR (*q*PCR). The *q*PCR amplification was performed for the functional genes *narG*, *napA*, *nirS*, *nirK* and *nosZ*. Additionally, the bacterial 16S rRNA gene was also quantified and used as a proxy for bacterial abundance. All reactions were performed in a 7500 Real Time PCR system (Applied Biosystems) using the SYBR® Green PCR Mastermix (Applied Biosystems). The reactions were performed with a 20 µl final volume containing 1X SYBR® Green Master Mix, 1 µg/µl BSA, 10 ng of DNA, and 1 µM of each primer. All primers were obtained from Biomers (Table S1).

The standard curves were generated using serial dilutions (from 10^2^ to 10^9^ copies/reaction) of plasmids containing known sequences of the targeted genes. The PCR efficiency ranged between 87.0 and 104.9%. The negative controls resulted in undetectable values in all *q*PCR reactions. An inhibition test was performed before the *q*PCR assays were done. Every sample was evaluated for inhibition independently. To detect possible inhibitory effects, 10^5^ copies of the plasmid DNA (pGEM®-T Easy, Promega, Madison, WI) were mixed with 10 ng of sample DNA and quantified with plasmid specific primers T7 and SP6. In all samples, the number of copies of the plasmid compared to the 16S rRNA concentration was sufficiently low (2 orders of magnitude) to detect significant variations of cycle thresholds (Ct) in case of inhibition [19]. In all cases Ct values when the plasmid was measured alone differed for less than 0.08±0.23 when compared with values obtained in inhibition tests. No significant differences in the inhibition tests were observed between samples of the three periods.

The relative contributions of the functional genes (*narG*, *napA*, *nirS*, *nirK* and *nosZ*) compared with the16S rRNA gene were calculated as a proxy for denitrifying bacteria abundance. The gene abundances and gene ratios were log transformed to ensure a normal distribution of the data. The normality was assessed for all variables, except for the abundances of the *narG* and *nirK* genes, using the Shapiro-Wilk tests. One-way ANOVA and post hoc tests (Tukey) were used with log-transformed data to characterise the effects of feeding regimes applied to the cathode on the abundance of different genes and ratios when equal variance of data was observed. Alternatively, non-parametric analyses (Kruskal-Wallis test) were used. All statistical analyses were performed using SPSS for Windows 15.0 (SPSS, Inc).

### Cloning of Functional Genes and Phylogenetic Analysis

The PCR products for the *narG*, *napA, nirS*, *nirK* and *nosZ* genes were purified using the QIAquick PCR purification kit (Qiagen, Germany) and cloned using the TOPO TA Cloning® Kit for Sequencing (Invitrogen, Eugene, OR) according to manufacturer’s instructions. At least 300 clones per gene were individually selected and screened by PCR using the primers M13 (Table S1). Amplicons of the expected size were sequenced at Macrogen (Macrogen, the Netherlands).

The sequences were examined for chimeras using the UCHIME algorithm [30] and manually refined using the BioEdit v7.0. The reference sequences for the *narG*, *napA*, *nirS*, *nirK* and *nosZ* genes were obtained from completely sequenced genomes in the GenBank database, aligned using CLUSTALW [31] and used as a template file to define Operational Taxonomic Units (OTUs) using Mothur v.1.22.1 [32]. OTUs were defined at threshold values of 33% (*narG*), 18% (*nirS*), 17% (*nirK*) and 20% (*nosZ*) [33], [34]. The threshold cut-off values for *napA* were set at 21%. This value was estimated as the species level cut-off value according to pair-wise comparisons of *napA* and 16SrRNA gene sequences of 23 completed genomes deposited in the GenBank database (results not shown) using a previously described computation procedure [33]. Defined OTUs were used to calculate rarefaction curves and estimate the richness (Chao1) and diversity indices (Shannon). Deduced amino acid sequences were obtained for the representative sequences of each OTU and functional gene and aligned using the ClustalW algorithm in MEGA v.5.0 [35]. Phylogenetic trees were reconstructed by neighbour-joining using the pair-wise deletion and p-distance methods. The tree topology was evaluated using bootstrap analysis with 10,000 replicates. Differences in the community composition based on the phylogeny of the *narG*, *napA*, *nirS*, *nirK* and *nosZ* genes were analysed from the tree topologies using a weighted Unifrac test [36].

### Nucleotide Sequence Accession Numbers

The sequence data derived in this study have been submitted to the GenBank database under the accession numbers JX236709 - JX236736 (*napA* gene), JX236737 - JX236898 (*narG* gene), JX237055 - JX237212 (*nirS* gene), JX236899 - X237054 (*nirK* gene) and JX237213 - JX237355 (*nosZ* gene).

## Results

### Denitrifying Cathode Performances Under Different Feeding Conditions

The MFC operated for one year under different cathodic feeding periods: autotrophic with nitrate (Period 1), heterotrophic with nitrate (Period 2) and autotrophic with nitrite (Period 3). Table 2 summarises the different performances of the MFC at those conditions. During the experimental study either nitrate or nitrite were removed in the cathode and a current was subsequently produced. The highest current production (15 A·m^−3^ NCC) and cathode Coulombic efficiencies (CE, 85%) were achieved when nitrate was used as the electron acceptor under strictly autotrophic conditions (Period 1). When organic matter was also introduced into the cathode (Period 2), electrotrophy was affected, decreasing the current production (11 A·m^−3^ NCC) and cathode Coulombic efficiency (58%). Nitrate was partially removed via conventional heterotrophic denitrification (using organic matter instead of electrons from the anode as the electron donor). The use of nitrite as an electron acceptor without the presence of organic matter resulted in an increase in current production (14.1 A·m^−3^ NCC) but cathode Coulombic efficiency was decreased (41%).

**Table 2:** Effect of different feeding conditions on the effluent characteristics of the cathode and the MFC performance.

Period	Flow (L·d^−1^)	COD (mg COD·L^−1^)	NO_3_^−^ (mg N-NO_3_^−^·L^−1^)	NO_2_^−^ (mg N-NO_2_·L^−1^)	N_2_O (mg N-N_2_O·L^−1^)	N_2_ (mg N-N_2_·L^−1^)	Cd (A·m^−3^ NCC)	CE (%)
**Period 1** (Autotrophic with NO_3_^−^)	2.0±0.3	n.d.	22.6±0.3	0.2±0.1	0.6±0.2	3.5±1.2	15.0±2.3	85±11
**Period 2** (Heterotrophic with NO_3_^−^)	1.4±0.5	31±22	20.7±4.8	2.1±3.4	4.0±1.9	3.8±1.8	11.0±7.0	58±17
**Period 3** (Autotrophic with NO_2_^−^)	1.2±0.2	n.d.	0.0±0.2	4.6±4.8	13.0±5.9	5.6±2.6	14.1±8.2	41±17

Concentrations of N_2_O and N_2_ were estimated on the basis of the electron balance at the cathode according to the method proposed of Virdis *et al*. [28].

NO_3_^−^, nitrate; NO_2_^−^, nitrite; N_2_O, nitrous oxide; N_2_, dinitrogen gas; Cd, current density; CE, coulombic efficiency.

The decrease of the CE suggested the presence of denitrification intermediates (nitrite or nitrous oxide) in the effluent. Table 2 shows the concentrations of the intermediates in the effluent of the cathode. As expected, nitrous oxide was produced at higher rates during Period 3. In this period, up to 70% of the nitrogen removed was converted into nitrous oxide. In contrast, when nitrate was feed during period 1, nitrous oxide level accounted for only 14% of the nitrogen removed.

### Quantification of the *narG, napA, nirK, nirS, nosZ* and 16S rRNA Genes

**Figure 1:**
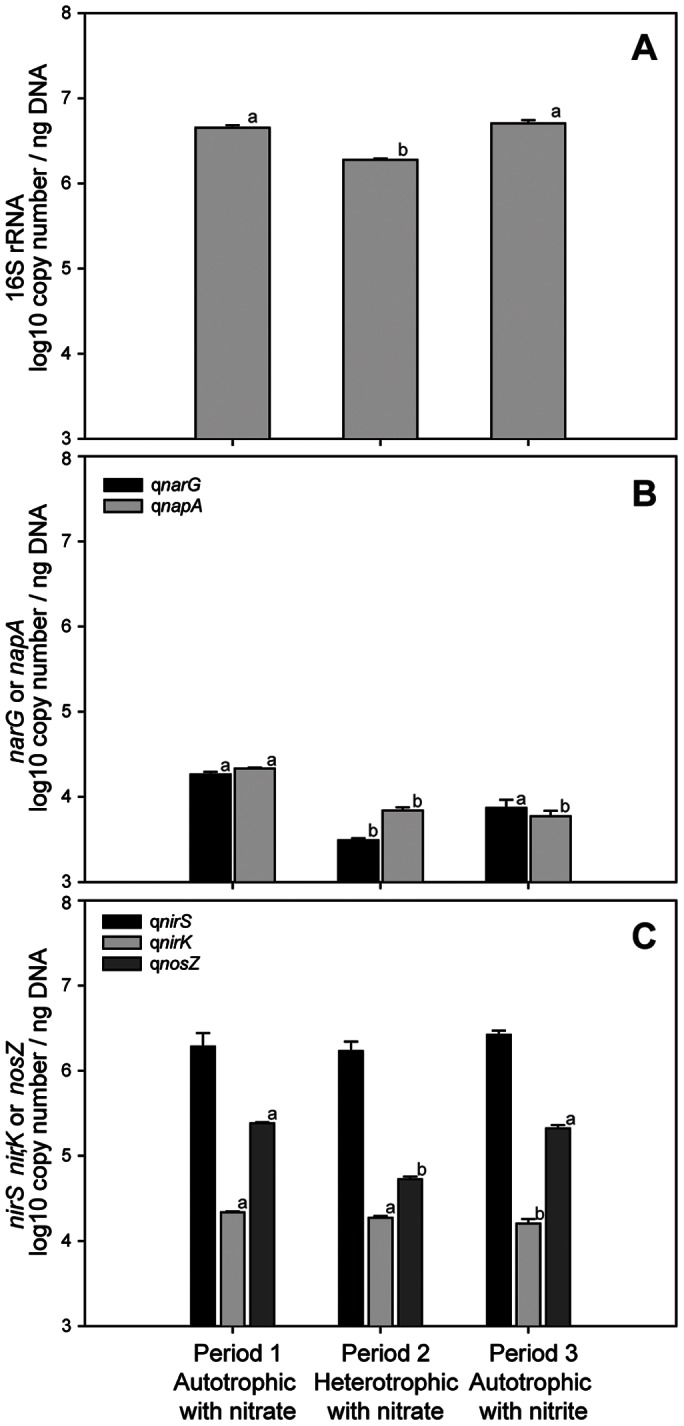
Abundances of the 16S rRNA and functional genes for the three periods studied. Mean values of the number of copies of the bacterial 16S rRNA (A) and the functional genes, *narG* and *napA* (B), *nirS*, *nirK* and *nosZ* (C) were represented according to the feeding periods in the cathode of a MFC. Standard errors of the mean are indicated. Different letters above the bars indicate significant differences (*p*<0.05) between periods.

The abundance of the 16S rRNA gene varied between 1.89×10^6^ and 5.07×10^6^ copies/ng DNA and was significantly higher (*p*<0.001) than most of the functional genes analysed (Figure 1). During the heterotrophic period, the abundance of 16S rRNA gene was significantly lower (*p*<0.026) compared to the autotrophic periods. The abundance of all functional genes varied between 0.3±0.0×10^4^ and 2.2±0.1×10^4^ gene copies/ng DNA, except for *nirS* and *nosZ*, which were detected at higher concentrations. The amount of *nirS* varied from 171.0±48.4×10^4^ to 264.8±32.0×10^4^ gene copies/ng DNA, but no significant differences were observed between periods. On the contrary, the abundance of *narG*, *napA*, *nirK* and *nosZ* showed significant differences (*p*<0.005) according to the operating conditions.

The relative contribution of the functional genes (*narG*, *napA*, *nirS*, *nirK* and *nosZ*) compared with the16S rRNA gene varied according to the feeding conditions (Table 3). The q*narG*/q16S rRNA ratio was significantly higher (*p*<0.01) when nitrate was used as the sole electron acceptor. The q*nirK*/q16S rRNA ratio was significantly different at all conditions tested and higher during the heterotrophic period. The q*nirS*/q16S rRNA ratio varied from 0.4 to 0.9, which were higher during the heterotrophic period (*p*<0.05). The q*nosZ*/q16S rRNA varied from 0.028 to 0.054 and was significantly lower (*p*<0.026) during heterotrophic growth.

**Table 3:** Relationship between the functional gene copy numbers, the 16SrRNA gene copy and the ratios between the genes.

Gene ratio	Period 1 (Autotrophic with NO_3_^−^)	Period 2 (Heterotrophic with NO_3_^−^)	Period 3 (Autotrophic with NO_2_^−^)	
q*narG*/*q*16S rRNA (×100)	0.4±0.1	0.2±0.0	0.1±0.0	ns
q*napA*/*q*16S rRNA (×100)	0.5±0.1	0.4±0.0	0.1±0.0	**
q*nirS*/*q*16S rRNA (×100)	42.0±15.9	90.0±23.9	52.2±3.4	*
q*nirK*/*q*16S rRNA (×100)	0.5±0.0	1.0±0.0	0.3±0.0	***
q*nosZ*/*q*16S rRNA (×100)	5.4±0.2	2.8±0.3	4.2±0.7	**
(q*narG*+q*napA*)/q*nosZ*	0.17±0.01	0.19±0.01	0.06±0.01	***
(q*narG*+q*napA*)/(q*nirS*+q*nirK*)	0.02±0.01	0.01±0.00	0.01±0.00	*
(q*nirK*+q*nirS*)/q*nosZ*	7.99±3.29	32.45±8.88	12.80±2.10	**

The values show the mean and standard deviation (SD) of two independent measurements. The statistical significances between the treatments were calculated using one-way ANOVA or Kruskal-Wallis test depending on the normality of the data. ns, not significant;

* *p*<0.05;

** *p*<0.01;

*** *p*<0.001.

The gene ratios were used to evaluate the capacity of the system to reduce nitrate to nitrogen gas during the sequential process of denitrification. (q*narG*+q*napA*)/q*nosZ* and (q*narG*+q*napA*)/(q*nirS*+q*nirK*) were consistently below a value of one, indicating a lower amount of nitrate reductases compared with the other steps in the denitrification pathway (Table 3). Significant differences of both ratios were observed in relation to feeding conditions. The (q*nirS*+q*nirK*)/q*nosZ* ratio varied from 7.99 to 32.45, indicating a great potential for the accumulation of intermediate gases.

### Community Structure of Denitrifying Bacteria

A total of 619 sequences, 162 for *narG*, 158 sequences for *nirS*, 156 for *nirK* and 143 for *nosZ*, were obtained from the cloning assay and used in the present study. Unfortunately, only 28 high-quality sequences could be obtained for the *napA* gene, although the cloning effort included the screening of 490 clones, which were too few to obtain an adequate analysis of the *napA*-containing community, thus the results have only been included for comparison with other genes.

The diversity and phylogenetic analyses were conducted on the basis of operational taxonomic units (OTUs). Rarefaction curves were constructed to visualise the saturation of the bacterial diversity (Figure S1). Except for the *napA* gene, the coverage values for all samples were higher than 90%, indicating that a representative portion of the bacterial diversity was covered (Table 4). The maximum richness (number of defined OTUs) was estimated according to Chao1 and varied from 5 to 32. Maximum values were identified for *nirS*-containing denitrifiers. The higher complexity of the *nirS*-containing community was confirmed from estimates of the Shannon diversity index. In contrast, the lowest diversity was observed for nitrous oxide reductase (*nosZ*) under all conditions.

**Table 4:** The values for the richness and diversity are shown.

Gene	Period 1 (Autotrophic with NO_3_^−^)					Period 2 (Heterotrophic with NO_3_^−^)					Period 3 (Autotrophic with NO_2_^−^)				
	n	S_obs_	S_Chao1_	C (%)	H’	n	S_obs_	S_Chao1_	C (%)	H’	n	S_obs_	S_Chao1_	C (%)	H’
***narG***	50	4	5	96	0.84±0.19	57	6	6.5	96.5	1.11±0.24	55	5	5.5	96.4	0.67±0.28
***napA***	9	6	16	88.9	1.58±0.57	11	5	6.5	90.9	1.29±0.53	8	3	4	75	0.74±0.59
***nirS***	60	15	25.5	93.3	2.29±0.22	42	16	34	92.9	2.45±0.26	56	19	22.5	96.6	2.63±0.23
***nirK***	54	12	17	93.6	1.57±0.36	54	15	18.3	96.3	2.40±0.22	48	9	9.6	100	1.19±0.38
***nosZ***	51	5	5.5	98.0	0.57±0.30	54	5	8	96.4	0.48±0.28	37	4	7	97.3	0.37±0.32

The mean and SD values are calculated for H’.

n, number of sequences used; S_obs,_ observed richness; S_Chao1,_ expected richness; C, coverage; H′, Shannon diversity index.

**Figure 2:**
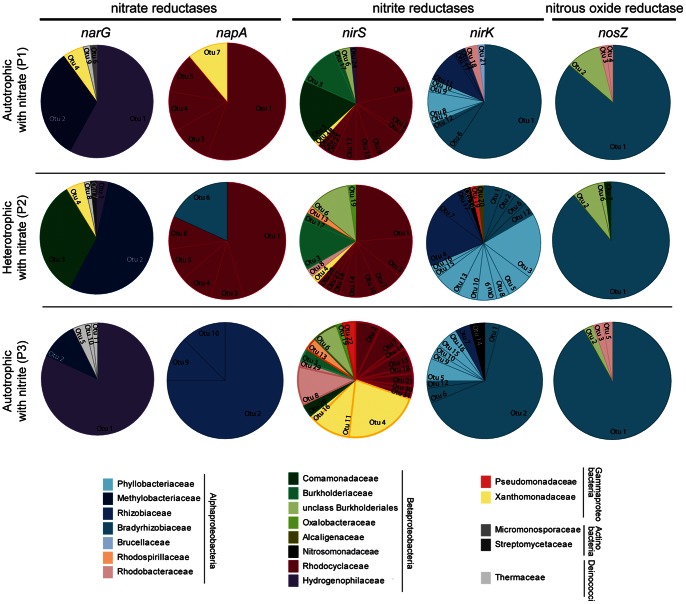
Composition of denitrifier communities. Pie charts comparing the community composition of denitrifiers according to functional gene similarities. Five denitrification genes, *narG*, *napA*, *nirS*, *nirK* and *nosZ*, at three different feeding periods in the cathode of a MFC are indicated. To facilitate the comparison between OTU distributions of bacterial communities, colors have been used as an indication of bacterial families and orders. Assignations have been made according to sequence similarities and may not reflect the exact phylogeny of the bacteria present.

The *narG* gene sequences were grouped into 11 different OTUs. OTU 1 and 2 were the most abundant, which comprised 76 and 53 sequences, respectively. OTU 1 was almost exclusively found during the autotrophic periods, whereas OTU 2 was predominantly found during the heterotrophic period (Figure 2). The OTU 1 and 2 representative sequences were approximately 81% similar to the betaproteobacterium *Thiobacillus denitrificans* and the alphaproteobacterium *Methylobacterium nodulans*, respectively. OTU 3 (19 sequences) was exclusively observed during the heterotrophic period and showed a low sequence similarity with most cultivated bacteria. Maximum similarities (73%) were observed with *Polaromonas naphthalenivorans* (Figure S2). The sequences of the periplasmic nitrate reductase (*napA*) were distributed into 10 different OTUs. The most abundant OTU (10 sequences) was shared between autotrophic and heterotrophic periods supplemented with nitrate and showed the highest sequence similarity (85%) to *Dechlorosoma suillum* (Figure S3). OTU 2 was similar to *Sinorhizobium fredii* (78%) and was exclusively observed during the autotrophic period with nitrite.

The *nirS* sequences were assigned to 31 different OTUs without a clear dominance. The most abundant OTUs, 1, 2 and 3, were affiliated with betaproteobacteria and showed relatively high similarities (>84%) with *Dechlorosomonas* sp., *Thauera* sp. and *Cupriavidus pauculus*, respectively. OTU 1 was exclusively observed under nitrate feeding conditions (Figure S4) whereas OTUs 2 and 3 were found at all operating conditions. OTUs 4, 8 and 11were observed almost exclusively during nitrite feeding conditions. According to BLAST searches with the reference genomic sequences database, the highest similarities of these sequences were found with the alphaproteobacterium *Paracoccus denitrificans* (OTU 8) and the gammaproteobacterium *Rhodanobacter* sp. (OTUs 4 and 11).

The gene encoding the copper-containing nitrite reductase, *nirK,* showed a different distribution between samples. Two out of a total of 22 OTUs were clearly dominant (up to 70 sequences) during autotrophic periods supplemented with nitrate and nitrite. The representative sequences of OTUs 1 and 2 were similar to *Sinorhizobium fredii* (84%) and *Rhodopseudomonas palustris* (85%), respectively (Figure S5). In contrast, during the heterotrophic period, *nirK* sequences distributed into 18 different OTUs. The most abundant OTU (82% similar to *Mesorhizobium* sp. 4FB11) comprised only 10 sequences.

The OTU distribution of *nosZ* genes revealed a relatively homogenous community for all periods. Almost 90% of sequences were grouped into a single OTU with a relatively high similarity (85%) to the predicted nitrous oxide reductase gene of *Oligotropha carboxidovorans* (Figure S6).

The significance of the observed differences between the microbial communities under different operating conditions in the MFC, was analysed using pair-wise weighted UniFrac analysis for the 5 molecular markers (Table 5). The UniFrac values confirmed the observed differences in the community composition, although statistically significant differences were only observed for *napA* and *nirS* containing communities. In both cases, significant differences were observed for the community of the autotrophic with nitrite period compared to the other periods. Low UniFrac values were obtained in all pair-wise comparisons for the *nosZ* community indicating a highly similar and stable community in all operating periods.

**Table 5:** Unifrac distance scores and *p* values of the denitrifier communities according to different functional genes of the three periods analysed in the MFC.

Gene	Period 1 *vs*.Period 2	Period 1 *vs*.Period 3	Period 2 *vs*.Period 3
***narG***	0.25 ns	0.13 ns	0.36 ns
***napA***	0.07 ns	0.41*	0.40*
***nirS***	0.10 ns	0.25*	0.22***
***nirK***	0.18 ns	0.22 ns	0.16 ns
***nosZ***	0.02 ns	0.04 ns	0.04 ns

The Unifrac values were calculated using amino acid deduced sequences and phylogenetic trees.

Period 1, Autotrophic with nitrate; Period 2, Heterotrophic with nitrate; Period 3, Autotrophic with nitrite; ns, not significant;

* *p*<0.05;

** *p*<0.01;

*** *p*<0.001.

## Discussion

### Influence of Cathode Feeding Characteristics on the Denitrifying MFC Performance

The power production and efficiency of nitrogen removal were influenced by the three cathodic influents (nitrate, nitrate plus organic matter and nitrite). The highest current production (15 A·m^−3^ NCC) was achieved when nitrate was used as the electron acceptor. In contrast, when organic matter was added, heterotrophic denitrification was kinetically favoured over autotrophic denitrification, and current production was reduced to 11 A·m^−3^ NCC. The use of nitrite as the initial electron acceptor without the presence of organic matter increased the current production to 14.1 A·m^−3^ NCC. Bacteria gain energy by transferring electrons from a reduced substrate at a low potential to an electron acceptor with a higher potential. The nitrate reduction potential (E°′ = +0.433 V *vs*. standard hydrogen electrode, SHE) is higher than that for nitrite (E°′ = +0.350 V *vs*. SHE) [3], therefore cathodic denitrification from nitrate is thermodynamically favourable under autotrophic conditions. The amount of energy available for the bacteria to grow in the cathode compartment using nitrate is estimated to be an 11% higher than nitrite.

The use of nitrate was not only beneficial for energy production, but also for the minimisation of nitrous oxide production, a gas with a strong greenhouse effect, and the increase of the maximum Coulombic efficiency. Apart from N_2_ production, N_2_O represents a major sink during nitrate removal in the MFC (Table 2). The reasons for the changing potential of N_2_O emissions are multifactorial and include differences in the prevailing physicochemical conditions (*i.e.* temperature, pH and C/N ratio), the nature of the main electron acceptor and the composition of the denitrifying community [37], [38], [39]. Under conditions of low electron availability (*i.e.* in the presence of organic matter), a lower affinity of the N_2_O reductase towards the electron donor facilitates the accumulation of this intermediate [28]. In addition, other studies suggest that the carbon source significantly impacts on the net N_2_O emission but exhibits a relatively minor effect on N_2_O production [40]. When nitrite was used as an electron acceptor in the denitrifying MFC, the Coulombic efficiency decreased to a 41%, compared to nitrate, and the N_2_O concentration in the effluent increased to 13 mg N-N_2_O·L^−1^ (a 70% of produced gases). It was proven that the use of nitrite as an electron acceptor and its accumulation in biological wastewater treatments cause an increase of NO and N_2_O emissions during denitrification, agreeing with the results obtained in the MFC [41], [42].

### Influence of the Cathode Feeding Characteristics on Denitrifier Communities

The MFC set-up provided excellent conditions to assess how changes in the main electron acceptors (nitrate *vs*. nitrite) and donors (cathode *vs*. organic matter) affected the composition of the denitrifier community and how it was related to the MFC performance. In the present study the PCR primers used are biased towards detecting mainly *Proteobacteria* (*nirK* and *nirS*) and the newly called Clade I *nosZ* gene, thus underestimating the actual diversity and abundance of nitrate and nitrous oxide reducers [39], [43], [44]. However, previous works analysing the bacterial diversity on MFC cathodes have shown these groups as particularly dominant in the biofilm community thus minimizing the impact of primer biases [11], [13]. Analysis of functional denitrification genes may reflect discrepancies, especially at the species-level, with the phylogeny of bacteria due to horizontal gene transfer and gene duplication events that may have occurred during evolution, and taxonomic inferences have to be taken cautiously [43]. The abundance of *narG* and *napA* containing nitrate reducers increased during nitrate addition thus showing the importance of these communities in the first reduction step. Changes in the abundance were accompanied by changes in the OTU composition. A large number of the retrieved *narG* sequences during autotrophic treatments supplemented with either nitrate or nitrite showed a high similarity to *narG* of the obligate chemolithoautotrophic bacterium *Thiobacillus denitrificans*. *T. denitrificans* has an optimal pH for growth around 7.5–8.0 and high denitrification rates (0.78 g NO_3_^−^ g cell^−1^·h^−1^) [45], which fall in the same range of those estimated in the MFC according to bacterial abundances. During the heterotrophic treatment, in contrast, analyses of the *narG* containing community revealed a higher relative abundance of sequences related to *Methylobacterium nodulans*, a bacterium able to grow using one carbon compounds and reducing nitrate to nitrite [46], and *Polaromonas naphthalenivorans,* a facultative chemolithotroph found in polluted habitats [47]. These two bacteria partially substituted obligate autotrophs during heterotrophic conditions.

The *nirS*-containing community showed highest similarities when nitrate was used as an electron acceptor despite the addition of organic matter. *nirS* sequences similar to those found in members of the family *Rodocyclaceae* were the most abundant. *Rodocyclaceae* have been found as the dominant bacterial population in industrial WWTPs [48]. OTU 6, with a high similarity to *Rubrivivax gelatinosus nirS* gene, accumulated during heterotrophic conditions. *Rubrivivax gelatinosus* has been described as an obligate nitrite reducer able to use different carbon sources [49]. In contrast, when nitrite was used as the first electron acceptor, *nirS* sequences similar to those found in Gammaproteobacteria, in particular *Rhodanobacter* sp., were the most abundant. A recent analysis of complete genome sequences of six *Rhodanobacter* strains isolated from soils have revealed that at least three of them lack the ability to reduce nitrate [50]. Similarly to what has been observed with the *nirS* gene, bacteria enriched when nitrite was used suggest the exclusive use of nitrite as electron acceptor. This is the case for OTU 2 (85% similar to *nirK* sequence of *Rhodopseudomonas palustris*). *Rhodopseudomonas palustris* lacks an ortholog of a dissimilatory nitrate reductase in its genome, suggesting that nitrate reduction cannot be done in this bacterium [51]. The addition of nitrite as initial electron acceptor impacted the composition of *nirS*- and *nirK-*type denitrifiers in the MFC, and possibly caused an enrichment of selected obligate nitrite reducers in view of sequence similarities with the detected functional genes.

Contrasting to the previous genes, the *nosZ*-containing community remained almost invariable during all conditions. Sequences with a high similarity to the *nosZ* gene of *Oligotropha carboxidovorans*, a carboxidotrophic bacterium [52], clearly dominated the *nosZ* community. The presence of *Oligotropha* like *nosZ* sequences has also been detected as major components of the nitrous oxide reducing communities in samples of acidic peat soils [34] and in the cathode of a denitrifying MFC [4]. Moreover, gene abundances during autotrophic conditions supported the idea of *nosZ* community minimally affected by the initial electron acceptor.

### Denitrifiers Affect N_2_O Accumulation in the MFC

Denitrification is considered the main source of N_2_O accumulation at a global scale [53], [54], [55], [56], [57]. However, no consensus exists whether to consider either the *nirS* or the *nirK* community as the main responsible for N_2_O accumulation, since the abundance of these two type denitrifiers can vary significantly from environment to environment [56], [58], [59], [60]. In the cathode of the MFC, NirS-type denitrifiers outnumbered NirK-type denitrifiers by two orders of magnitude at all working conditions. High q*nirS*/16S rRNA values were observed, suggesting a clear implication of the *nirS*-type denitrifiers in the denitrification potential and the accumulation of N_2_O.

Different alternatives were considered to explain the high q*nirS/*q16S rRNA ratio found. First, the presence of multiple copies of *nirS* gene in a single genome, *i.e*. *Thauera* sp., *Thiobacillus denitrificans*, *Dechloromonas aromatica* or *Magnetospirillum magneticum* [43], [61], was evaluated. Second, an overestimation of the *nirS* abundance due to *q*PCR bias was considered. However, this possibility was excluded after the examination of dissociation curves and the cloning of random *q*PCR products, which led us to confirm the specificity of the reaction [19]. The observed prevalence of *nirS* over *nirK* denitrifiers may be the result of a selective enrichment of the former due to a putative enhanced capacity of electron harvesting by cytochrome *c* family mediators. This feature has been confirmed in *Geobacter sulfurreducens* ATCC 51573 by the analysis of a GSU3274 deletion mutant. GSU3274 is a gene coding for a putative cytochrome *c* family protein [62].

Despite the occurrence of some limitations, such as the presence of multiple gene copies per genome and differences in specific activity [17], [43], *q*PCR analyses of functional genes provide significant data to infer community dynamics [18], [63]. The ratio between the abundance of nitrite reductases and nitrous oxide reductase allowed us to estimate the potential to reduce completely nitrite to N_2_. In the MFC and at the working conditions used in this study, the estimated N_2_O accumulation significantly correlated (r^2^ = 0.992) with the (q*nirK*+q*nirS*)/q*nosZ* ratio. Higher accumulations of N_2_O were observed when nitrite was used as the electron acceptor, similarly to what has been described in other environments [34], [42].

In conclusion, the cathodic biofilm of the MFC was dominated by *nirS*-type denitrifiers at all conditions tested and its abundance relative to nitrous oxide reducers highly correlated with N_2_O emissions. The denitrifying bacterial communities identified affected the electrochemical performance increasing the current density for about 25% in autotrophic conditions. Also the suspected relevant players in nitrate and nitrite reduction have been identified on the basis of functional gene similarities. Their relative dominance at each period was highly affected by the changes of the electron acceptor or electron donors. Contrarily, the *nosZ* community remained almost invariable during all periods tested. Obtained *nosZ* sequences showed a high similarity to nosZ gene of *Oligotropha carboxidovorans*, suggesting that may have an active and preponderant role in electron harvesting in the cathode surface. This may raise new questions, such as which mechanisms are involved in electron transfer and what the location of *O. carboxidovorans*-like bacteria in the biofilm is, that will be investigated in the near future.

## Supporting Information

Figure S1 Rarefaction curves. Rarefaction curves calculated for each gene and period. Cut-off values for OTU definitions were 33% for *narG* gene, 21% for *napA* gene, 18% for *nirS* gene, 17% for *nirK* gene and 20% for *nosZ* gene.
(TIF)

Figure S2 *narG* phylogenetic tree. Neighbor-joining phylogenetic tree of amino acid deduced *narG* sequences. The representative sequences of each OTU and accession numbers of deposited sequences are shown. The percentage of sequences from the three conditions analysed are indicated (P1, Autotrophic with nitrate; P2, Autotrophic with nitrite; P3, Heterotrophic with nitrate). The bootstrap values higher than 50% are shown at the nodes of the tree (10,000 replicates). The reference sequences were retrieved GenBank and added for comparison. *narG* gene of *Haloarcula marismortui* ATCC 43049 (NC_006397) was used as outgroup.
(TIF)

Figure S3 *napA* phylogenetic tree. Neighbor-joining phylogenetic tree of amino acid deduced *napA* sequences. The representative sequences of each OTU and accession numbers of deposited sequences are shown. The percentage of sequences from the three conditions analysed are indicated next to each OTU, (P1, Autotrophic with nitrate; P2, Autotrophic with nitrite; P3, Heterotrophic with nitrate). The bootstrap values higher than 50% are shown at the nodes of the tree (10,000 replicates). The reference sequences were retrieved from GenBank and added for comparison. *napA* gene of *Escherichia coli* ATCC8739 (CP000946) was used as outgroup.
(TIF)

Figure S4 *nirS* phylogenetic tree. Neighbor-joining phylogenetic tree of amino acid deduced *nirS* sequences. The representative sequences of each OTU and accession numbers of deposited sequences are shown. The percentage of sequences from the three conditions analysed are indicated next to each OTU (P1, Autotrophic with nitrate; P2, Autotrophic with nitrite; P3, Heterotrophic with nitrate). The bootstrap values higher than 50% at the nodes of the tree (10,000 replicates). The reference sequences were retrieved from GenBank and added for comparison. *nirS* gene of *Rhodothermus marinus* DSM 4252 (NC_013501) was used as outgroup.
(TIF)

Figure S5 *nirK* phylogenetic tree. Neighbor-joining phylogenetic tree of amino acid deduced *nirK* sequences. The representative sequences of each OTU and accession numbers of deposited sequences are shown. The percentage of sequences from the three conditions analysed are indicated next to each OTU (P1, Autotrophic with nitrate; P2, Autotrophic with nitrite; P3, Heterotrophic with nitrate). The bootstrap values higher than 50% are shown at the nodes of the tree (10,000 replicates). The reference sequences were retrieved from GenBank and added for comparison. *nirK* gene of *Nitrosomonas* sp. C-56 (AF339044) was used as outgroup.
(TIF)

Figure S6 *nosZ* phylogenetic tree. Neighbor-joining phylogenetic tree of amino acid deduced *nosZ* sequences. The representative sequences of each OTU and accession numbers of deposited sequences are shown. The percentage of sequences from the three conditions analysed are indicated next to each OTU (P1, Autotrophic with nitrate; P2, Autotrophic with nitrite; P3, Heterotrophic with nitrate). The bootstrap values higher than 50% are shown at the nodes of the tree (10,000 replicates). The reference sequences were retrieved from GenBank and added for comparison. *nosZ* gene of *Haloarcula marismortui* ATCC 43049 (AY596297) was used as outgroup.
(TIF)

Table S1 Primers and conditions used for PCR.
(DOCX)

References S1 References added to Table S1. List of references that were included in TableS1 but not in the main text file. The numbering follows the one used in the main text.
(DOCX)
