# Supplementary figures and images for: Denitrifying Bacterial Communities Affect Current Production and Nitrous Oxide Accumulation in a Microbial Fuel Cell
Source: PLoS One. 2013 May 23;8(5):e63460. doi: 10.1371/journal.pone.0063460 (PMC3662693; doi:10.1371/journal.pone.0063460)

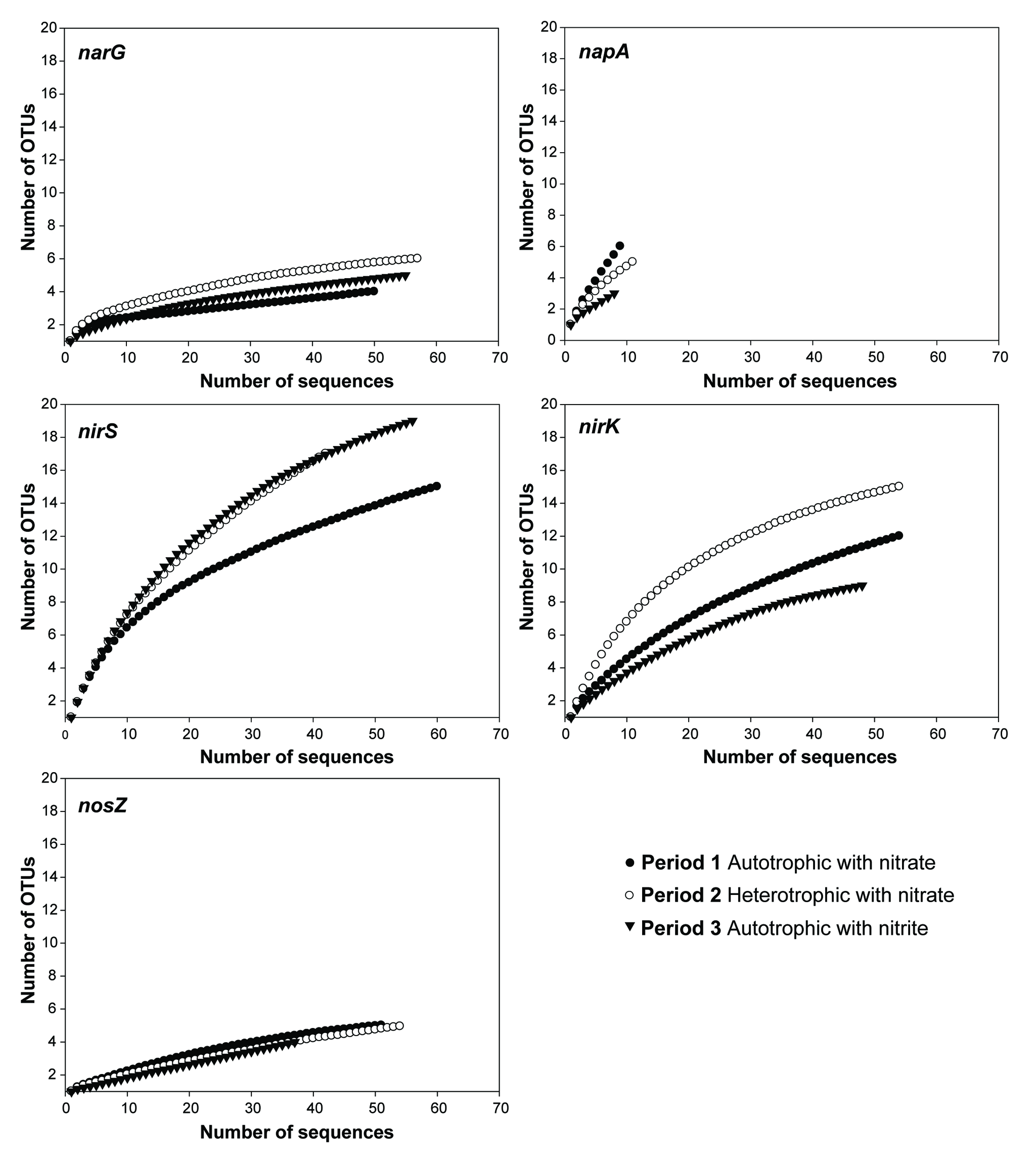

Supplement: Figure S1 — Rarefaction curves. Rarefaction curves calculated for each gene and period. Cut-off values for OTU definitions were 33% for narG gene, 21% for napA gene, 18% for nirS gene, 17% for nirK gene and 20% for nosZ gene. (TIF) [file pone.0063460.s001.tif]

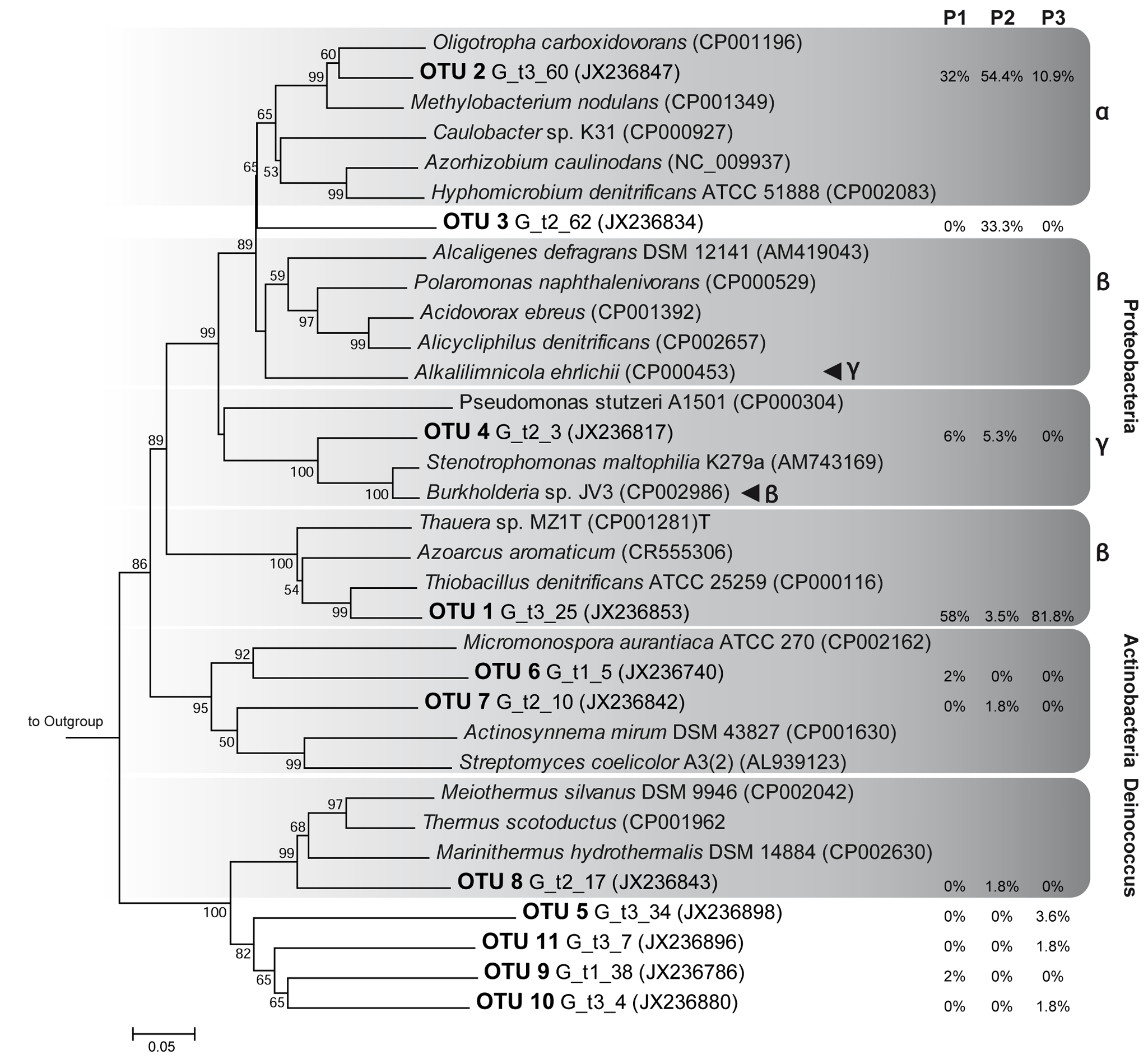

Supplement: Figure S2 — narG phylogenetic tree. Neighbor-joining phylogenetic tree of amino acid deduced narG sequences. The representative sequences of each OTU and accession numbers of deposited sequences are shown. The percentage of sequences from the three conditions analysed are indicated (P1, Autotrophic with nitrate; P2, Autotrophic with nitrite; P3, Heterotrophic with nitrate). The bootstrap values higher than 50% are shown at the nodes of the tree (10,000 replicates). The reference sequences were retrieved GenBank and added for comparison. narG gene of Haloarcula marismortui ATCC 43049 (NC_006397) was used as outgroup. (TIF) [file pone.0063460.s002.tif]

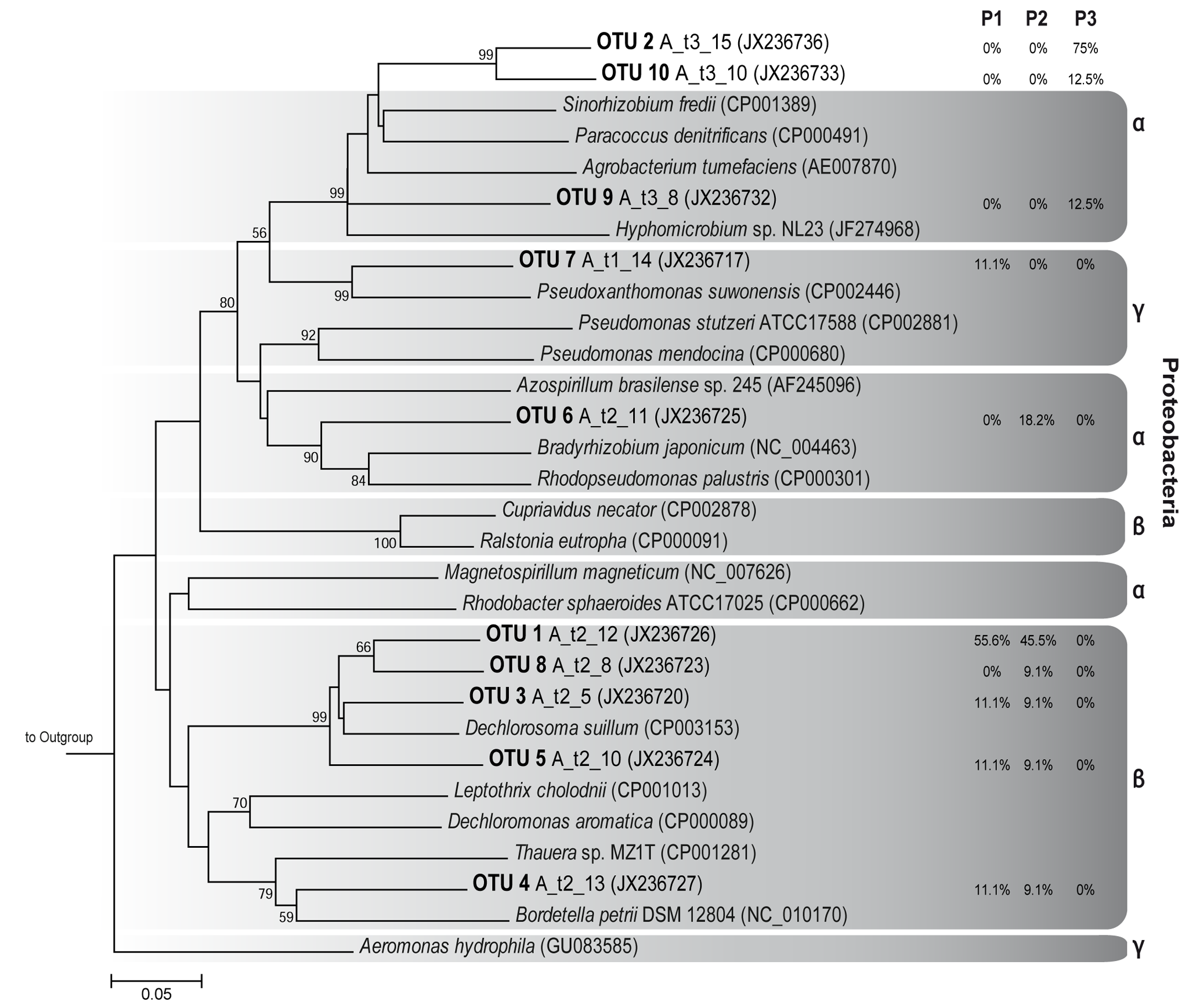

Supplement: Figure S3 — napA phylogenetic tree. Neighbor-joining phylogenetic tree of amino acid deduced napA sequences. The representative sequences of each OTU and accession numbers of deposited sequences are shown. The percentage of sequences from the three conditions analysed are indicated next to each OTU, (P1, Autotrophic with nitrate; P2, Autotrophic with nitrite; P3, Heterotrophic with nitrate). The bootstrap values higher than 50% are shown at the nodes of the tree (10,000 replicates). The reference sequences were retrieved from GenBank and added for comparison. napA gene of Escherichia coli ATCC8739 (CP000946) was used as outgroup. (TIF) [file pone.0063460.s003.tif]

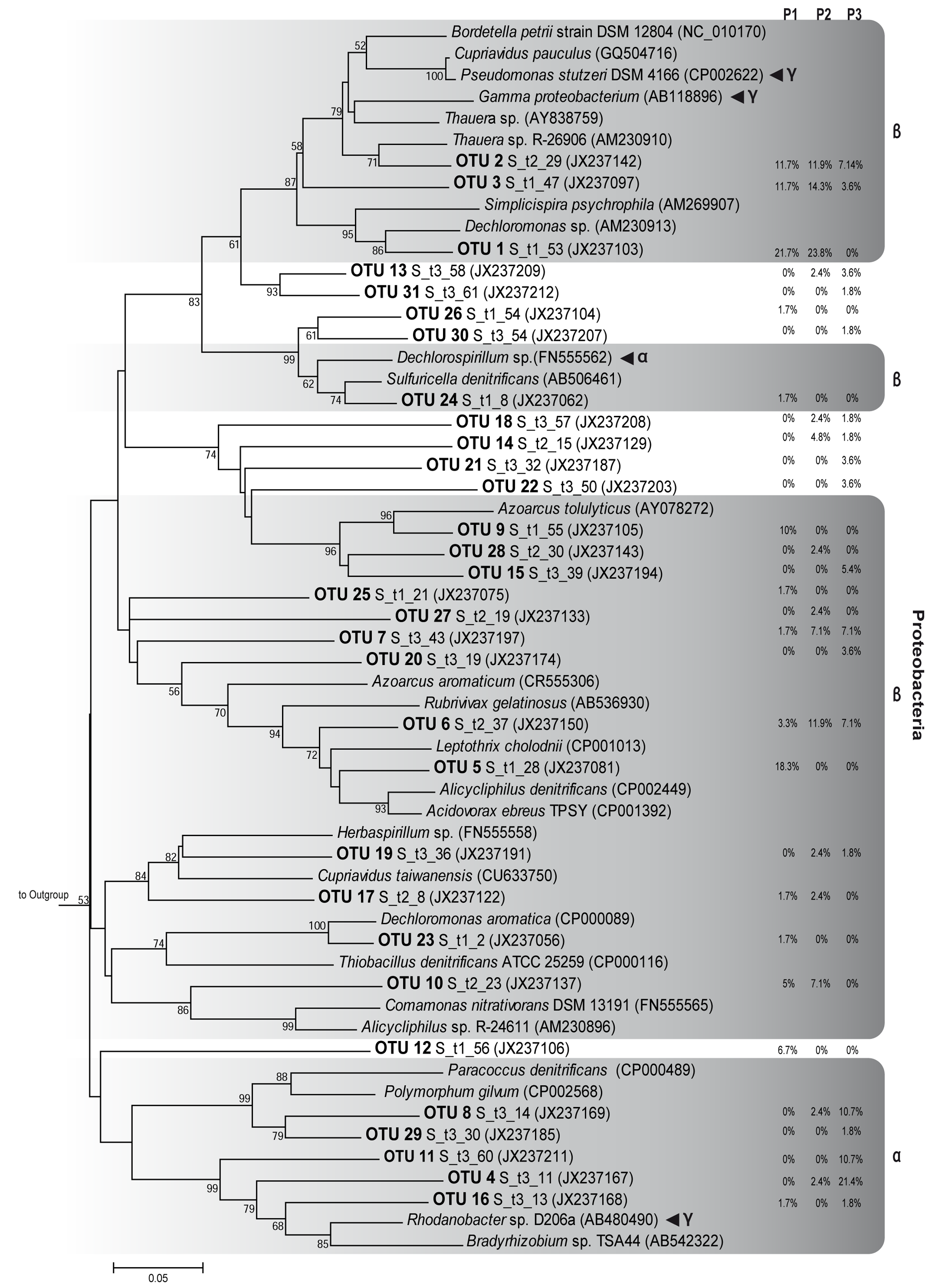

Supplement: Figure S4 — nirS phylogenetic tree. Neighbor-joining phylogenetic tree of amino acid deduced nirS sequences. The representative sequences of each OTU and accession numbers of deposited sequences are shown. The percentage of sequences from the three conditions analysed are indicated next to each OTU (P1, Autotrophic with nitrate; P2, Autotrophic with nitrite; P3, Heterotrophic with nitrate). The bootstrap values higher than 50% at the nodes of the tree (10,000 replicates). The reference sequences were retrieved from GenBank and added for comparison. nirS gene of Rhodothermus marinus DSM 4252 (NC_013501) was used as outgroup. (TIF) [file pone.0063460.s004.tif]

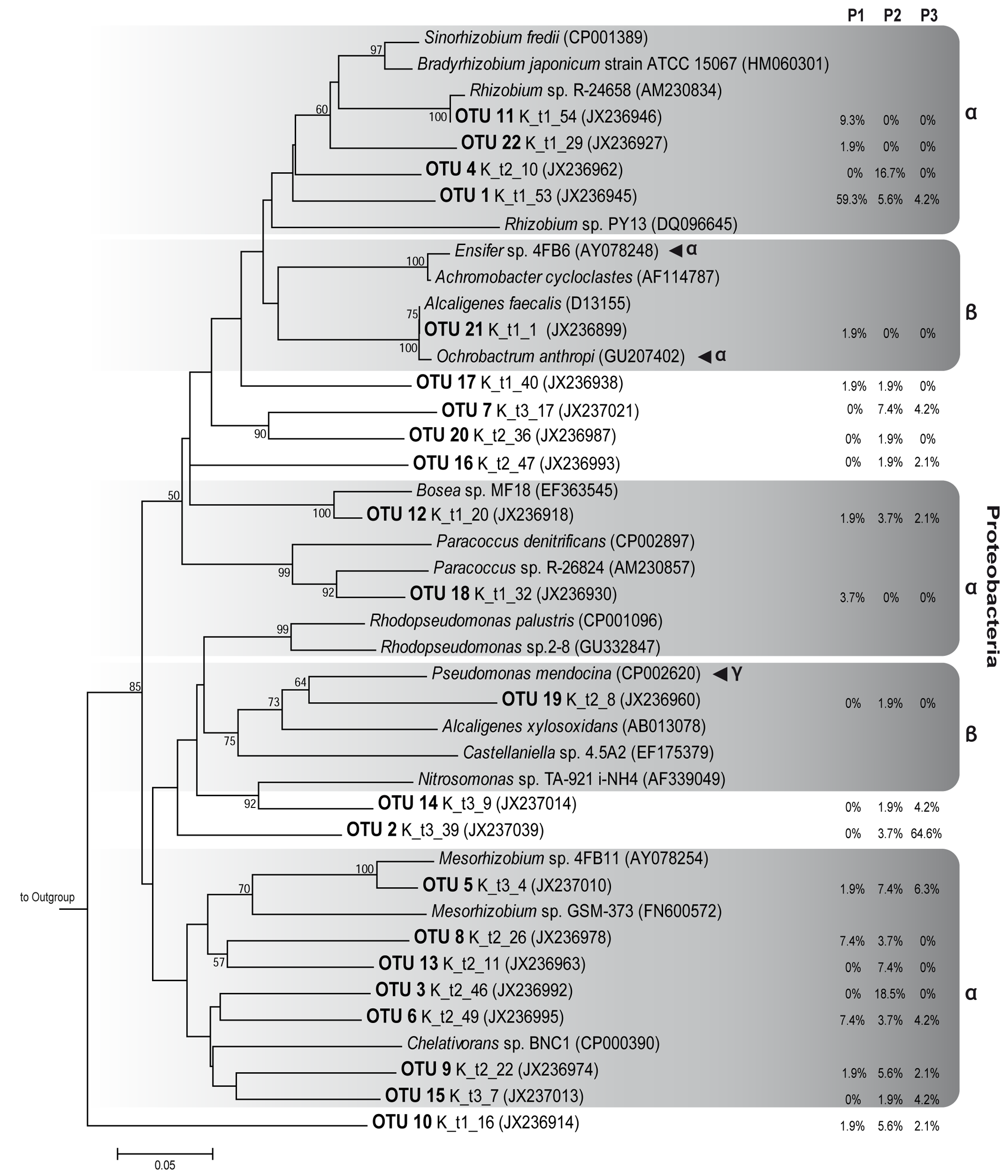

Supplement: Figure S5 — nirK phylogenetic tree. Neighbor-joining phylogenetic tree of amino acid deduced nirK sequences. The representative sequences of each OTU and accession numbers of deposited sequences are shown. The percentage of sequences from the three conditions analysed are indicated next to each OTU (P1, Autotrophic with nitrate; P2, Autotrophic with nitrite; P3, Heterotrophic with nitrate). The bootstrap values higher than 50% are shown at the nodes of the tree (10,000 replicates). The reference sequences were retrieved from GenBank and added for comparison. nirK gene of Nitrosomonas sp. C-56 (AF339044) was used as outgroup. (TIF) [file pone.0063460.s005.tif]

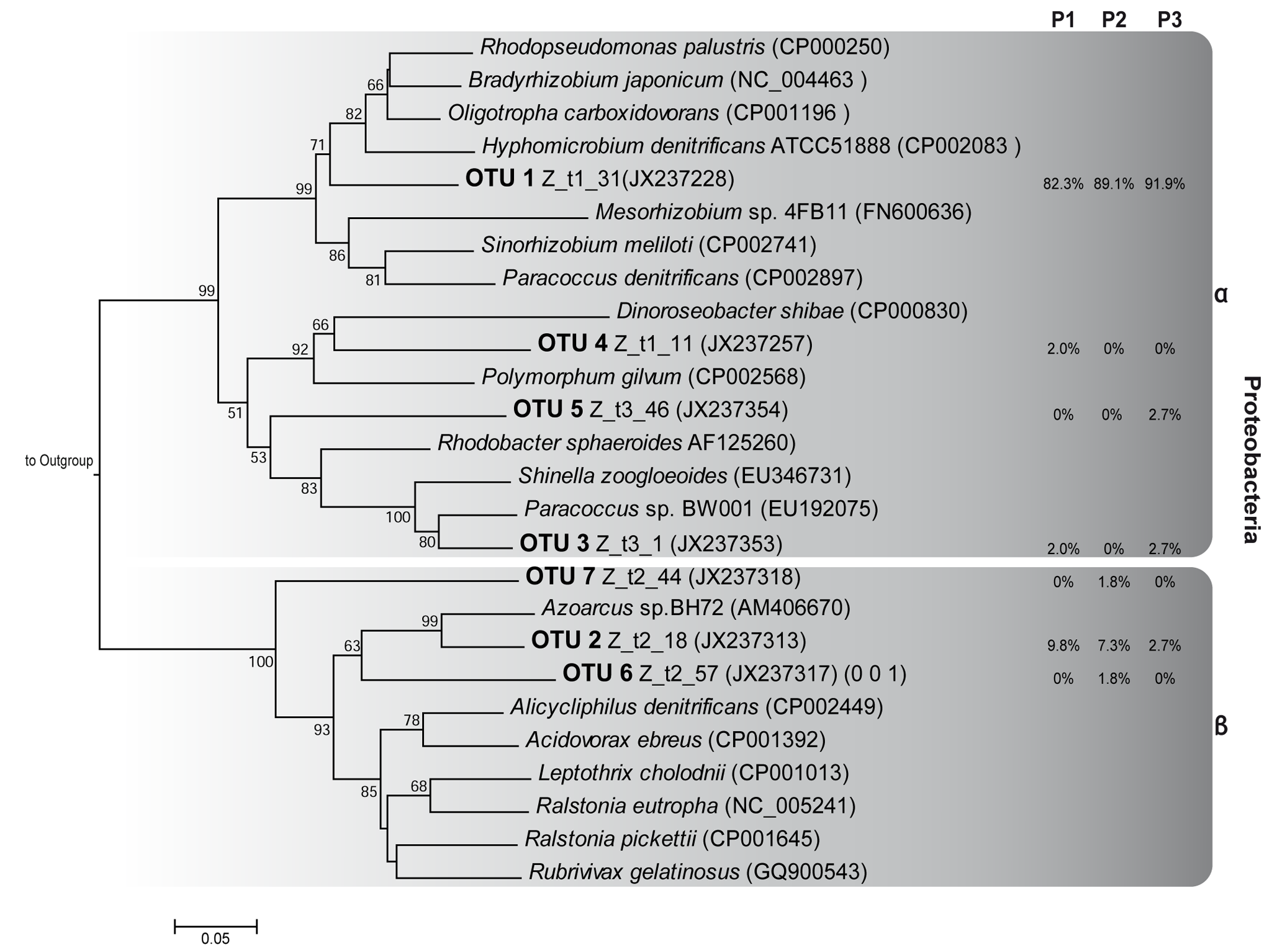

Supplement: Figure S6 — nosZ phylogenetic tree. Neighbor-joining phylogenetic tree of amino acid deduced nosZ sequences. The representative sequences of each OTU and accession numbers of deposited sequences are shown. The percentage of sequences from the three conditions analysed are indicated next to each OTU (P1, Autotrophic with nitrate; P2, Autotrophic with nitrite; P3, Heterotrophic with nitrate). The bootstrap values higher than 50% are shown at the nodes of the tree (10,000 replicates). The reference sequences were retrieved from GenBank and added for comparison. nosZ gene of Haloarcula marismortui ATCC 43049 (AY596297) was used as outgroup. (TIF) [file pone.0063460.s006.tif]
