# Supplementary material for: Denitrifying Bacterial Communities Affect Current Production and Nitrous Oxide Accumulation in a Microbial Fuel Cell
Source: PLoS One. 2013 May 23;8(5):e63460. doi: 10.1371/journal.pone.0063460 (PMC3662693; doi:10.1371/journal.pone.0063460)
Supplement: Table S1 — Primers and conditions used for PCR. (DOCX) [file pone.0063460.s007.docx]

| Gene | Primers | Sequence (5’ – 3’) | Amplicon (bp) | PCR type | PCR conditions^a^ | Reference |
| --- | --- | --- | --- | --- | --- | --- |
| 16S rRNA | 341F | CCT ACG GGA GGC AGC AG | 194 bp | *q*PCR |  | [19,64] |
|  | 534R | ATT ACC GCG GCT GCT GGC A |  |  |  |  |
| narG | narG-F | TCGCCSATYCCGGCSATGTC | 173bp | *q*PCR |  | [65] |
|  | narG-R | GAGTTGTACCAGTCRGCSGAYTCSG |  |  |  |  |
|  | narG1960F | TAYGTSGGSCARGARAA | 650 bp | PCR | Primer conc: 0.5 μM | [17] |
|  | narG2650R | TTYTCRTACCABGTBGC |  |  |  |  |
| napA | napA-V17 | TGGACVATGGGYTTYAAYC | 152bp | *q*PCR |  | [65] |
|  | napA4r | ACYTCRCGHGCVGTRCCRCA |  |  |  |  |
|  | napV67m | AAYATGGCVGARATGCACCC | 514bp | PCR |  | [66] |
|  | napV17m | GRTTRAARCCCATSGTCCA |  |  |  |  |
| **nirS** | nirSCd3aFm | AAC GYS AAG GAR ACS GG | 425bp | *q*PCR |  | [15,19] |
|  | nirSR3cdm | GAS TTC GGR TGS GTC TTS AYG AA |  |  |  |  |
|  | cd3aF | GTSAACGTSAAGGARACSGG | 425 bp | PCR | Primer concentration: 0.25 μM, Annealing temp: 57ºC | [15,67] |
|  | R3cd | GASTTCGGRTGSGTCTTG |  |  |  |  |
| **nirK** | nirK876 | ATY GGC GGV CAY GGC GA | 164bp | *q*PCR |  | [19,20] |
|  | nirK1040 | GCC TCG ATC AGR TTR TGG TT |  |  |  |  |
|  | F1aCu | ATCATGGTSCTGCCGCG | 472 bp | PCR | Primer conc: 1.0 μM, Annealing temp: 60ºC, BSA: 400 ng/μl | [19] |
|  | R3Cu | GCCTCGATCAGRTTGTGGTT |  |  |  |  |
| **nosZ** | nosZ2F | CGC RAC GGC AAS AAG GTS MSS GT | 267b | *q*PCR |  | [19,20] |
|  | nosZ2R´ | CAK RTG CAK SGC RTG GCA GAA |  |  |  |  |
|  | *nosZ*-F | CGYTGTTCMTCGACAGCCAG | 453 bp | PCR | Primer concentration: 0.8 μM, BSA: 800 ng/μl, Qiagen Q Solution: 1X | [15,68] |
|  | *nosZ*1622R | CGSACCTTSTTGCCSTYGCG |  |  |  |  |
| **Others** | M13F-20 | GTAAAACGACGGCCAG | -- | PCR |  | TOPO TA Cloning® Kit for Sequencing (Invitrogen, Eugene, OR) |
|  | M13R | CAGGAAACAGCTATGAC |  |  |  |  |
|  | SP6 | ATTTAGGTGACACTATAG | -- | PCR |  | pGEM® Vectors Promega (Promega Biotech Ibérica) |
|  | T7 | TAATACGACTCACTATAGGG |  |  |  |  |

^a^ Only changes from the original PCR conditions are indicated. BSA: Bovine Serum Albumin. Quantitative reactions are shown as *q*PCR
