# Supplementary material for: Denitrifying Bacterial Communities Affect Current Production and Nitrous Oxide Accumulation in a Microbial Fuel Cell
Source: PLoS One. 2013 May 23;8(5):e63460. doi: 10.1371/journal.pone.0063460 (PMC3662693; doi:10.1371/journal.pone.0063460)
Supplement: References S1 — References added to Table S1. List of references that were included in TableS1 but not in the main text file. The numbering follows the one used in the main text. (DOCX) [file pone.0063460.s008.docx]

**References added to Table S1**. List of references that were included in TableS1 but not in the main text file. The numbering follows the one used in the main text.

64. López-Gutierrez JC, Henry S, Hallet S, Martin-Laurent F, Catroux G, et al. (2004) Quantification of a novel group of nitrate-reducing bacteria in the environment by real-time PCR. J Microbiol Methods 57: 399-407.

65. Bru D, Sarr A, Philippot L (2007) Relative abundances of proteobacterial membrane-bound and periplasmic nitrate reductases in selected environments. Appl Environ Microbiol 73: 5971-5974.

66. Henry S, Texier S, Hallet S, Bru D, Dambreville C, et al. (2008) Disentangling the rhizosphere effect on nitrate reducers and denitrifiers: insight into the role of root exudates. Environ Microbiol 10: 3082-3092.

67. Michotey V, Mejean V, Bonin P (2000) Comparison of methods for quantification of cytochrome *cd_1_*-denitrifying bacteria in environmental marine samples. Appl Environ Microbiol 66: 1564-1571.

68. Kloos K, Mergel A, Rösch C, Bothe H (2001) Denitrification within the genus *Azospirillum* and other associative bacteria. Aust J Plant Physiol 28: 991-998.
